# Dissociation Constant of Integrin-RGD Binding in Live Cells from Automated Micropipette and Label-Free Optical Data

**DOI:** 10.3390/bios11020032

**Published:** 2021-01-24

**Authors:** Tamás Gerecsei, Péter Chrenkó, Nicolett Kanyo, Beatrix Péter, Attila Bonyár, Inna Székács, Balint Szabo, Robert Horvath

**Affiliations:** 1 Centre for Energy Research, Nanobiosensorics Laboratory, Institute of Technical Physics and Materials Science, 1121 Budapest, Hungary; gerecsei.tamas@mfa.kfki.hu (T.G.); pajtas1.29@gmail.com (P.C.); kanyo.nikolett@energia.mta.hu (N.K.); peter.beatrix@energia.mta.hu (B.P.); szekacs.inna@energia.mta.hu (I.S.); balint.szabo@ttk.elte.hu (B.S.); 2 Department of Biological Physics, Eötvös Loránd University, 1117 Budapest, Hungary; 3 Department of Electronics Technology, Budapest University of Technology and Economics, 1111 Budapest, Hungary; bonyar@ett.bme.hu

**Keywords:** adhesion, micropipette, waveguide, biosensor, two-dimensional dissociation constant, integrin-RGD-binding

## Abstract

The binding of integrin proteins to peptide sequences such as arginine-glycine-aspartic acid (RGD) is a crucial step in the adhesion process of mammalian cells. While these bonds can be examined between purified proteins and their ligands, live-cell assays are better suited to gain biologically relevant information. Here we apply a computer-controlled micropipette (CCMP) to measure the dissociation constant (*K_d_*) of integrin-RGD-binding. Surface coatings with varying RGD densities were prepared, and the detachment of single cells from these surfaces was measured by applying a local flow inducing hydrodynamic lifting force on the targeted cells in discrete steps. The average behavior of the populations was then fit according to the chemical law of mass action. To verify the resulting value of *K_d_*^2*d*^ = (4503 ± 1673) 1/µm^2^, a resonant waveguide grating based biosensor was used, characterizing and fitting the adhesion kinetics of the cell populations. Both methods yielded a *K_d_* within the same range. Furthermore, an analysis of subpopulations was presented, confirming the ability of CCMP to characterize cell adhesion both on single cell and whole population levels. The introduced methodologies offer convenient and automated routes to quantify the adhesivity of living cells before their further processing.

## 1. Introduction

Integrin-mediated cellular adhesion is one of the most fundamental processes in the life of a mammalian cell. For most cell types, adhesion to a surface is a prerequisite for survival and normal functioning. A family of heterodimer transmembrane receptors, integrins, are responsible for initiating and maintaining contact between the cell and its surroundings [1]. Besides acting as a molecular anchor, integrins react to physical cues and transfer signals from the environment to the inside of the cells. Namely, they are connected to the cytoskeleton (via additional proteins such as Arp2/3, vinculin, etc.), which acts as a channel of information between the outer membrane and the cell nucleus where most of the genetic regulatory processes take place [2,3]. The principal binding target of integrins is the arginine-glycine-aspartic acid (RGD) tripeptide motif, which is present on numerous extracellular matrix (ECM) proteins, such as laminin and fibronectin. Artificial polymers containing the RGD motif have been employed with great success to precisely tailor the cell adhesion properties of surfaces [4,5,6]. The ability of cells to spread and actively adhere to a substrate depends on the two-dimensional RGD density [7] and patterning [8]. Therefore, by tuning these parameters, a wide range of functionalized surfaces can be created from largely cell repellent to adhesion initiating. One widespread chemical method to achieve tight control over RGD surface density is the adsorption of RGD-functionalized PLL-g-PEG-RGD synthetic copolymers on a substrate. A PLL-g-PEG molecule consists of PLL (poly-L-lysine) chains that can adsorb to most electron donating substrates and PEG (polyethylene-glycol) chains that are exposed to the bulk over the surface, impeding adhesion of certain cell types [9] and proteins [10].

While the components of the glycocalyx can regulate the strength and kinetics of cellular adhesion [11], the PLL-g-PEG-RGD is reported to interact only with the integrins available at the bottom surface of the cells. Here, PEG chains are sufficiently long to effectively repel cells and to block any direct interactions between the positively charged PLL chain and the surface of the cells [12]. Of note, targeted experiments with varied PEG chain lengths could reveal the existence of such interactions.

Covalently grafting an RGD motif to such a polymer makes it possible to cover a surface with integrin ligands while effectively suppressing non-specific adsorption. Furthermore, mixing PLL-g-PEG and its RGD grafted version in different ratios allows for the precise control of the ligand-to-ligand distance in the formed layers, as has been shown by Orgovan et al. [13]. Promising results have also been attained using dextran hydrogels [14].

There are several methods for characterizing cell adhesion on such functionalized surfaces [15]. An emerging technique operating on the single-cell level is the computer-controlled micropipette [16,17], which is based on a glass micropipette connected to a pressure controller system. Adhered cells on a substrate are automatically directed under the micropipette, which attempts to aspire them using a negative pressure-induced flow. Measuring the negative pressure at which a given cell is removed from the surface, an adhesion distribution can be created. This way, it is not only possible to describe the behavior of populations, but also to identify subpopulations or even individual outlier cells. Since the micropipette is mounted on an inverted microscope, the user has total, label-free visual control over the sample. This allows for adhesion characteristics to be correlated to visual cues such as cell size and morphology. The micropipette-based method also entails an important versatility in the types of samples that can be examined. This includes various cell types [16], but also other non-biological samples such as functionalized microbeads [17] as well. The latter can be used as a colloidal force spectroscopy tool to investigate the characteristics of immobilized receptor–ligand pairs.

A general descriptor of such chemical systems is the dissociation constant (*K_d_*), which shows the concentration of free ligands at which half of the receptors are in a complex at binding equilibrium. Since the interaction of integrins with surface ligands is a process restrained in two dimensions during cellular adhesion, two-dimensional chemical parameters can be reasonably defined [13,18]. In such cases, the diffusivity of the receptor and the ligand can have a determining role, which is illustrated by the activation of mobility-dependent integrin activation pathways [19]. The two-dimensional equilibrium dissociation constant can be used to calculate its three-dimensional counterpart through division by a length characterizing the confinement zone at the interface, which reasonably corresponds to the average separation of the cell membrane and the substrate [20].

Dissociation constant measurements are common in binding assays using immobilized molecules of the receptor brought in contact with a solution of the ligands [21,22]. However, when both the ligand and the receptor are embedded into the membrane of a live cell, for example, in the case of an integrin complex, measuring *K_D_* values becomes challenging. Essentially, a choice must be made between using cell-based assays in order to preserve the native environment and thus functionality of integrins or using free-integrin-based assays [23]. The latter provides chemically precise and reproducible information that is truly specific to the integrin subtype used in the assay. However, measured values might be biologically irrelevant due to the tertiary structure of proteins being compromised upon removal from the cell membrane, as well as the inability of free integrins to form two-dimensional clusters in solution. It is important to note, measuring the binding in a more native system resulted in increased dissociation constant as summarized in Orgovan et al. [13]. In contrast, cell-based assays utilize the adhesion of live cells as an indication of ligand-binding, retaining integrin functionality. The special trait of this technique is that the expression level of integrins varies largely by cell type as well as in certain cases by time, external conditions, or regulatory processes [24]. In order to gain physiologically important information on integrin ligand-binding, live cell-based methods hold great promise as they have the capacity to include the entire biological response of cells that is relevant in an in vivo context [25]. Most assays utilize optical methods to assess cell attachment, such as coverage evaluation [26], immunostaining of focal adhesion proteins [27], or more elaborate kinetic biosensor measurements [13,28].

In the present paper, we demonstrate that the dissociation constant between membrane-bound integrin in live cells and RGD motifs can effectively be measured by a computer-controlled micropipette. By measuring cell adhesion on surfaces with various (two-dimensional) RGD surface densities, we could fit the value of two-dimensional *K_d_* based on simple theoretical considerations. The techniques elaborated here can be adapted for various cell–substrate, microbead-substrate or even microbead-cell interactions, providing a complete capability to quantitatively describe such systems.

## 2. Materials and Methods

### 2.1. Cell Culture and Seeding

HeLa cells used in the experiments (93021013 Sigma-Aldrich, St. Louis, MO, USA) were kept in Dulbecco’s Modified Eagle’s Medium (DMEM), with 10% fetal bovine serum (Biowest SAS, Nuaillé, France), 4 mM L-glutamine, 100 U/mL penicillin and 100 µg/mL streptomycin in an incubator with a humidified atmosphere containing 5% CO_2_ at 37 °C. Before micropipette experiments, cells were washed with Dulbecco’s phosphate-buffered saline (DPBS, Sigma-Aldrich, St. Louis, MO, USA ) and detached by applying 0.05% (*w/v*) trypsin and 0.02% (*w/v*) EDTA solution for 2 min in the incubator. Cells were resuspended in DMEM, and the concentration was measured using a Bürker counting chamber. HeLa cells with a density of 10^4^ cells per mL were pipetted in surface-coated Petri dishes. Subsequently, the Petri dishes containing the cells were incubated at 37 °C with 5% CO_2_ for 90 min to allow for adhesion.

### 2.2. Surface Coating

For surface coating the following polymers were used: poly(L-lysine)-graft-poly(ethylene glycol) (PLL-g-PEG, [PLL(20)-g(3.5)-PEG(2)]) (hereafter PP) and its RGD-grafted version:

PLL-g-PEG/PEGGGGGYGRGDSP (PLL-g-PEG-RGD [PLL(20)-g(3.5)PEG(2)/PEG(3.4)-RGD]) (hereafter PPR). Both materials were obtained in powder form from SuSoS AG (Dübendorf, Switzerland) and were stored at −20 °C until use. The molecular weight of the functionalized copolymers was *M_Pol_* = 107.76 kDa, the fraction of functionalized PEG chains was *P* = 14.7% and the grafting ratio g = 3.5. Eight different ratios of PPR to PP (*Q*) were used in live-cell experiments: *Q* = 1%, 2%, 3%, 5%, 10%, 25%, 50%, 100% with the last concentration being pure PPR. The PLL-g-PEG polymers were diluted to a concentration of 0.5 mg/mL in 10 mM HEPES at pH = 7.4 directly before use with a final volume of 400 µL. The coating solution was then pipetted on a glass-bottomed Petri dish (Greiner, Hungary), which was placed on a rocker for 30 min allowing for the polymer layer to form. Subsequently, the coating solution was removed, and the dish was washed three times with 10 mM HEPES. Following the washing steps, the 10 mM HEPES was removed, and the cells were added to the dish in DMEM solution. For the cell adhesive coatings, the molar surface density of RGD motifs was determined by the following equation [13]:

(1) $$\rho_{RGD}=\frac{\Gamma }{M_{Pol}}\frac{Q}{100}\frac{N_{Lys}}{g}\frac{P}{100}$$

where *Γ* = 97 ng/cm^2^ is the surface density adsorbed to the bare surface, and *N_Lys_* = 136.82 is the average number of lysines per PLL backbone. The average distance of RGDs (*d_RGD_*_−*RGD*_) can be expressed in the following form, supposing a hexagonal arrangement of the ligands (N_A_ is the Avogadro number):

(2) $$d_{RGD-RGD}=\sqrt{\frac{2}{\sqrt{3}}\frac{1}{\rho_{RGD}N_{A}}.}$$

### 2.3. Micropipette Measurements

**Figure 1:**
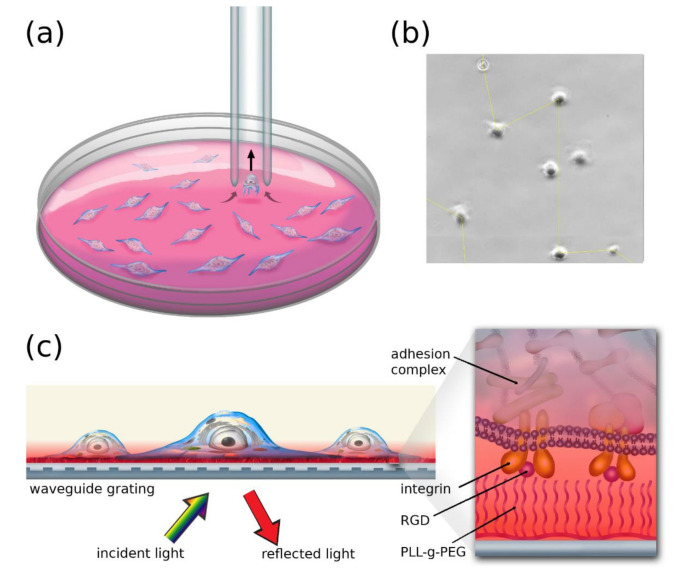
Illustration of the methods used to measure cell adhesion (**a**). The computer-controlled micropipette (CCMP) automatically visits each detected cell and probes them by applying a preset negative pressure. In the drawing, the targeted cell detaches from the surface and enters the micropipette. The ratio of cells picked up in a round is determined by scanning the area of interest with the optical microscope on which the CCMP is mounted. The size of the Petri dish and the cells are not to scale for better visibility (**b**). Section of a field of view in a Petri dish containing adhered HeLa cells. The yellow line shows the path of the micropipette that was automatically calculated by the CCMP control software. Note that the two cells that were too close to each other (closer than 70 µm) were excluded from the measurement. (**c**) Schematic representation of the resonant waveguide-based measurement. The cells adhere to the sensor plate, which is illuminated from below. The intensity of the reflected resonant light is detected by a charge-coupled device (CCD) camera (not shown). The magnified drawing shows the binding of integrins to the arginine-glycine-aspartic acid (RGD)-grafted PLL-g-PEG (PP) layer and the intracellular adhesion complex. Red coloring signifies the evanescent electric field, the intensity of which decreases exponentially into the media above the sensor. The refractive index change is measured within this field through the wavelength shift of the reflected light.

Cell adhesion measurements were executed with an imaging-based computer-controlled micropipette (CCMP) system (CellSorter, Budapest, Hungary). A schematic representation can be seen in Figure 1a. First, a Petri dish containing the adhered cells was placed onto the motorized stage of an inverted microscope (Zeiss Axio Observer Z1). A part of the surface was scanned then the cells were detected using a built-in algorithm [16]. In the case of two cells within 70 µm of each other, they were automatically excluded from the measurement in order to avoid probing multiple cells at the same time (Figure 1b). The remaining population contained 95–130 cells. After detection, a 70 µm inner diameter glass micropipette was automatically placed above each cell, and a preset negative pressure was applied for 25 ms to probe cellular adhesivity. The pressure was adjusted using a 60 mL syringe that was connected to the micropipette through a normally closed fluid valve. After the whole population was probed, the negative pressure was increased, and the remaining cells were revisited by the micropipette. During one measurement, five such rounds were completed, each with increasing negative pressure. After each round, the area of interest was scanned, and the coordinates of the detected cells were saved for analysis. Processing of the data was executed by a Python 3.0 script comparing the coordinates of detected cells before and after each round. The ratio of cells remaining on the surface after applying given negative pressure values was saved and analyzed further.

### 2.4. The Resonant Waveguide Grating (RWG) Imager Biosensor

The adhesion kinetics of HeLa cells was monitored using the Epic BT biosensor (Corning Inc., Glendale, CA, USA), which is an evanescent field-based resonant waveguide grating imager allowing high-throughput label-free detection at a solid–liquid interface (Figure 1c). In our measurements, a 384-well uncoated Epic microplate (5040, Corning) was used. On the bottom of each well, an optical grating was embedded, which coupled the resonant wavelength component of the incident laser light into the waveguide layer. The resonant wavelength depended on the refractive index of the liquid within the evanescent electric field generated by the total internal reflections of the light within the waveguide. The latter field had a penetration depth of 150 nm using the zeroth-order TM mode. The light was coupled out, and a CCD detector was used to measure its intensity. The shift of the resonant wavelength (∆*λ*, given in picometers) was the primary signal of RWG. Due to the spreading and adhering cells recruiting proteins in the close vicinity of the substrate, the wavelength shift signal was directly proportional to the adhesion force and adhesion energy [28].

Preparing the surface coating, each well of the 384-well microplate was hydrated with 30 µL of 10 mM HEPES buffer pH 7.4 for 20 min. Subsequently, 30 µL of the coating solution containing the mixture of PLL-g-PEG (PP) and PLL-g-PEG grafted with RGD (PPR) was pipetted into the wells. In order to eliminate any bubbles in the wells, the plate was centrifuged at 800× *g* for 10 s, then placed on a rocker for 30 min at room temperature. After adsorption of the polymer layer, wells were washed three times using 20 mM HEPES-HBSS buffer, then filled up with 20 µL of the same buffer. The baseline was recorded with the RWG biosensor for 15 min. Subsequently, 20 µL of cell suspension containing 8000 cells was added to the wells. Cell adhesion kinetics was monitored for 1.5 h.

## 3. Results

### 3.1. Cell-Based Dissociation Assay

In order to acquire the dissociation constant value of the RGD-integrin-binding in living HeLa cells, functionalized surfaces with various RGD surface densities were prepared. The micropipette measured the remaining population fractions after applying a stepwise increasing negative pressure. Three separate experiments were conducted with each PPL-g-PEG-RGD to PLL-PEG ratio (*Q*). These data can be processed in different ways. One approach is to take the average of the three independently measured population fractions at each point.

**Figure 2:**
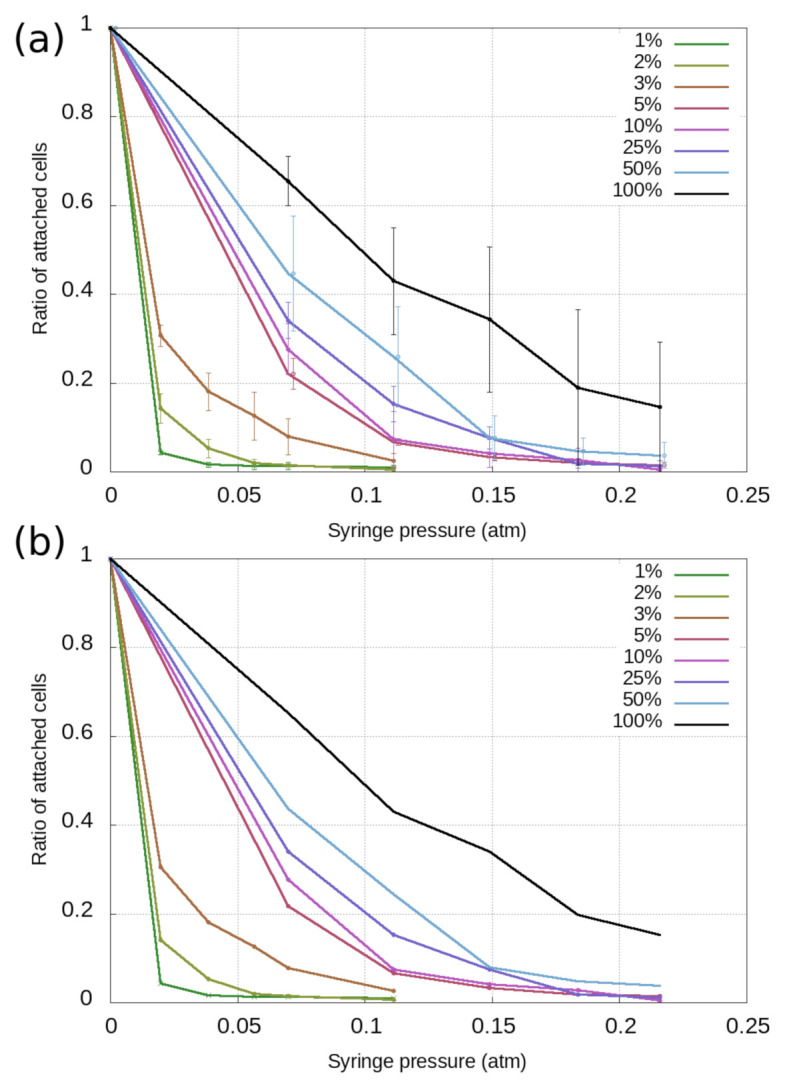
Curves representing the detachment of cells from surfaces with different PPR to PP ratios (*Q*). (**a**) Averaging of data: each point corresponds to the average of values measured in three separate experiments, while the error bars represent the standard error. (**b**) The pooling of data: points correspond to unified datasets coming from three separate experiments.

In Figure 2a, the averaged curves are presented with the standard error (standard deviation divided by the square root of the number of measurements) given as error bars.

Another approach is to consider all data measured using the same surface treatment (*Q*) to be part of the same population. In this case, we can pool the data together and simply add the number of remaining cells at each point, then divide them with the sum of the beginning populations. This approach gives us the detachment curves presented in Figure 2b. Note that in this case, there is no error in the data since the three measurements are regarded as one.

Let *x*_0_, *y*_0_, and *z*_0_ be the number of cells at the beginning of the three separate experiments and *x_n_*, *y_n_*, and *z_n_* the number of cells that remain on the surface after applying the *n*th negative pressure value. The ratio of cells remaining on the surface is then *R_a_* for averaging and *R_p_* for pooling:

(3) $$R_{a}=\frac{1}{3}\left(\frac{x_{n}}{x_{0}}+\frac{y_{n}}{y_{0}}+\frac{z_{n}}{z_{0}}\right)$$

(4) $$R_{p}=\frac{x_{n}+y_{n}+z_{n}}{x_{0}+y_{0}+z_{0}}$$

Regardless of the data evaluation scheme, it is observable that as the RGD content of the coating decreases, cells tend to detach from the surface at smaller negative pressure values. While all experiments were conducted under the same conditions, the random variation of cellular populations in their adhesion characteristics is better considered by the averaging method. The population diversity can then be quantified by the standard error of population fractions.

In order to quantitatively characterize the adhesion of the population as a whole, a numerical parameter should be introduced, which takes into account all the data contained within the detachment curves.

**Figure 3:**
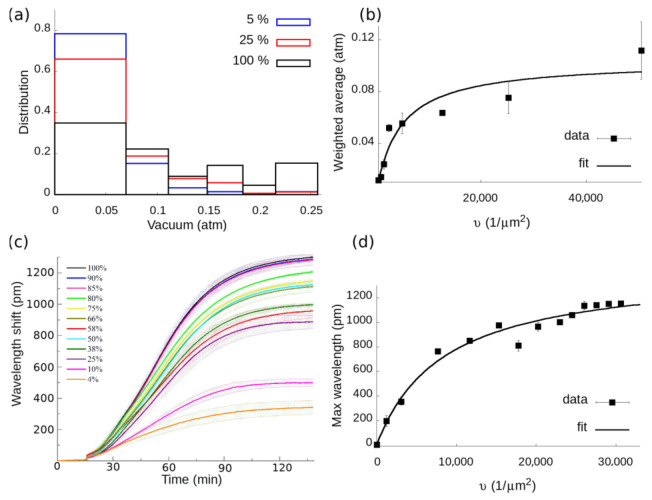
(**a**): Histograms of cell detachment calculated from the detachment curves. The inset shows the PPR to PP percentage ratio (*Q*) of the surfaces on which the cells were seeded. As *Q* increases, the distributions shift to the right, towards higher detachment pressures. (**b**) The fit of Equation (6) on the weighted average of all detachment histograms. From the fit, the two-dimensional dissociation constant can be determined. Error bars show the standard error of 3 measurements. (**c**) Adhesion kinetics of cell populations measured by RWG optical biosensor. Surface coatings with different PPR to PP ratio were used to create different RGD densities. The maximum wavelength shift was fitted for each curve according to Equation (8), and the resulting values were plotted against the RGD density in (**d**). Here, Equation (6) is fitted on the maximal wavelength values to determine the two-dimensional dissociation constant. Error bars show the standard error of three separate measurements.

First, let us consider the histograms that can be calculated from the detachment curves. The difference between two points (e.g., *x_n_* and *x_n_*_+1_) corresponds to the ratio of cells whose detachment pressure was in the corresponding interval ([*p_n_*_+1_,*p_n_*]). By plotting these ratio differences in bins, histograms can be generated (see Figure 3a).

Comparing distributions corresponding to different *Q* values shows that a higher RGD content causes the distribution to shift to the right. Since the mode of the histograms remains the same, namely the smallest negative pressure value, a peak-like parameter seems to be an insufficient descriptor to grasp the complexity of the distribution change. However, the weighted average of such histograms (*W*) is expected to increase with increasing *Q* and constitutes a quantitative measure of cell adhesion based on the measured data. By definition, the weighted average is the sum of the product of each bin’s value (*x_n_* − *x_n_*_+1_) and the middle of its interval $\left(p_{n}+\frac{p_{n+1}-p_{n}}{2}\right)$.

(5) $$W=\overset{N}{\underset{n=0}{\sum }}\left(x_{n}-x_{n+1}\right)\left(p_{n}+\frac{p_{n+1}-p_{n}}{2}\right)$$

where *N* is the number of pressure steps, in our case, 6. Indeed, the weighted average defined in this way increases with increasing RGD content of the surface, as seen in Figure 3b. Considering a steady-state in the frame of the kinetic mass action law (KMAL), the following formula can be fit to determine the two-dimensional equilibrium dissociation constant [13]:

(6) $$B=\frac{L_{0}I_{0}}{L_{0}+K_{d}^{2D}},$$

where *B* is the number of bound ligands and *L*_0_ is the surface concentration of available ligands corresponding to the two-dimensional RGD density (*ν*). A reasonable assumption is that the amount of bounds ligands is proportional to the degree of cell adhesion, which in this case is characterized by the weighted average of the histograms (*B*∼*W*).

The fit of Equation (6) on the weighted averages of all measured histograms is presented in Figure 3b. The two-dimensional dissociation constant is then $K_{d}^{2D}=4503\pm 1673\frac{1}{\mu m^{2}}$.

For comparison with other cell-based assays, the three-dimensional dissociation constant must be determined using the thickness of the confinement region (*l_c_*) as:

(7) $$K_{d}=K_{d}^{3D}=\frac{K_{d}^{2D}}{l_{c}}.$$

We assume *l_c_ =* 100 nm corresponding to the separation of the cell and the substrate [13]. Using Equation (7), the estimated value of the equilibrium dissociation constant is *K_d_* = (75 ± 28) µM.

### 3.2. Cell Adhesion on RGD-Tuned Surfaces with RWG Biosensor

In order to verify this result against an already established cell-based assay, we used an RWG optical biosensor, where the wavelength shift of the reflected resonant light is directly proportional to the adhesion force and adhesion energy of the surface attached cells [28]. Cells were attached to the RGD grafted PLL-g-PEG surfaces on the biosensor substrate, and their kinetic wavelength shift curves were recorded during the course of their attachment and spreading. The kinetic curves were fitted with the logistic equation according to:

(8) $$\frac{d\lambda }{dt}=r\lambda \left(1-\frac{\lambda }{\lambda_{max}}\right),$$

as seen in Figure 3c. The degree of adhesion in the steady-state is then characterized by the saturation value of the sigmoid (*λ_max_*), which acts as an analog of the histograms’ weighted average.

It is apparent that decreasing the distance of RGD motifs increases the maximal wavelength shift in accordance with previous results [11,13]. By fitting Equation (6) to the data under the assumption that *λ_max_*∼*B*, a value of $K_{d}^{2D}=8433\pm 1679\frac{1}{\mu m^{2}}$ is acquired, therefore $K_{d}^{3D}=140\pm 28\mu M$ (see Figure 3d). While this value is in the same order of magnitude as the one measured by the CCMP, there is a roughly two-fold mismatch. Note, the employed force exerted by the deadhesion process during CCMP measurements can also contribute to this difference.

However, considering such molecular parameters, it can be concluded that the equilibrium dissociation constants measured by the two methods are in reasonable agreement.

### 3.3. Analysis of Subpopulations

So far, we focused on analyzing the adhesion data on the level of the entire population. From the accurate measurement of the dissociation constant, it is demonstrated that CCMP can reproduce regular life cell assay capabilities. However, the histograms such as the ones in Figure 2a can be further analyzed to extract information on the nature of the inhomogeneity of the cell population. Namely, each bin can be regarded as a subpopulation made up of cells having adhesion forces in the given interval defined by the position and width of the bin. The lowest interval, for instance, represents weakly adhered cells that are detached by the micropipette during the first round using a negative pressure of 0.07 atm. Therefore, the “weakly attached” subpopulation is the fraction of the entire population, which is made up of only those cells that show detachment pressures in the 0–0.07 atm range. Similarly, strongly attached cells are defined as cells that were not detached even by the largest applied negative pressure of 0.22 atm. The micropipette collects information selectively from each of these subpopulations, therefore their behavior can be examined separately.

**Figure 4:**
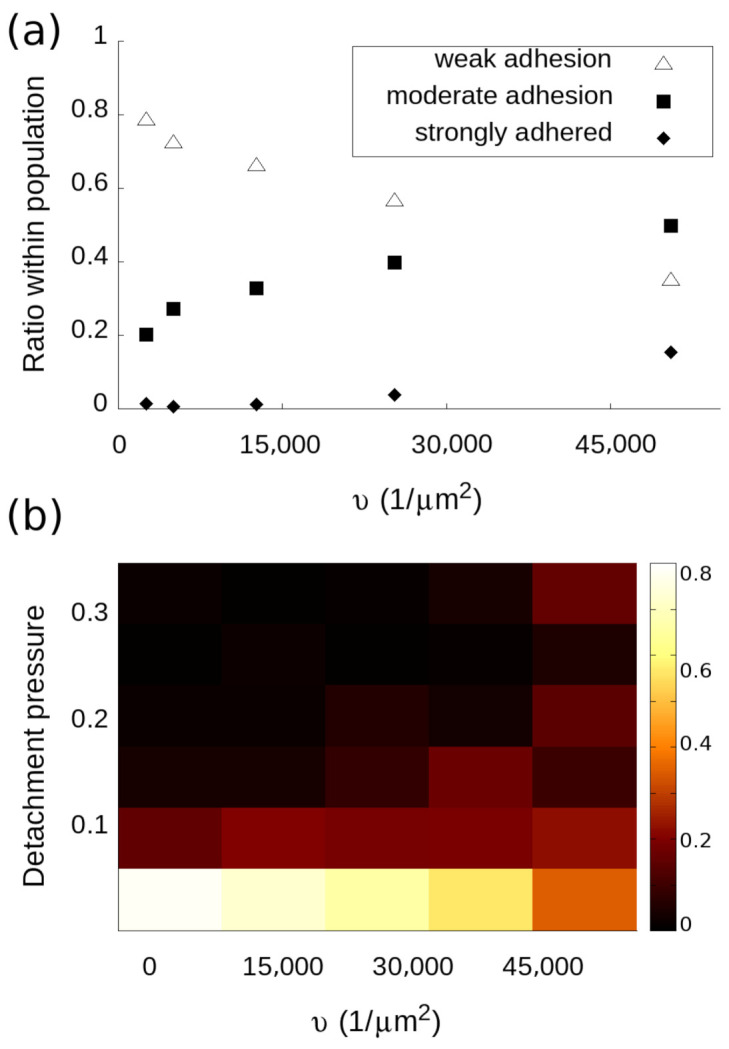
Subpopulation analysis of HeLa cells on different RGD densities. (**a**) Dependence of the fraction of weakly, moderately, and strongly adhered cells on the RGD density. Weak adhesion stands for cells that were picked up in the first round of probing by the negative pressure of 0.07 atm, while strongly adhered cells were not picked up even with a negative pressure of 0.22 atm. Moderate adhesion stands for cells in between the 0.07–0.22 atm range. (**b**) Heat map of the distribution of cell adhesion. The color scale indicates the ratio of cells having the indicated detachment pressure on the substrate with the given RGD density. It can be observed that the distribution widens and flattens with increasing ligand density.

In the case of RGD tuning, one would expect the strongly attached population to become more prominent as the RGD density is increased. At the same time, the ratio of weakly adhered cells is expected to decrease. As seen in Figure 4a, the ratio of weakly attached cells decreases from 80% to 35%, which means that even at a 100% PPR coating, one-third of the population exhibits weak adhesion. It is interesting to note that weakly adhered cells comprise the majority of the population up until reaching the highest RGD density. Conversely, the fraction of the population showing the strongest adhesion only becomes discernible later on when the RGD-RGD distance has increased above the critical separation. The fraction of the cell population in between (moderate adhesion) shows a dependence on RGD density resembling that of the weighted average of the histograms. It is evident that the change of population-level adhesion as a function of RGD cannot be thought of as the simple translation of a well-defined narrow distribution. Instead, a widening and spreading distribution in the adhesion space should be considered where the weighted average is shifting to higher adhesion values while a wide range of adhesion behaviors are present simultaneously. To further explore this changing distribution, a heat map can be created (Figure 4b). It can be observed that the adhesion distribution stays centered near the weak adhesion interval. Interestingly, measuring the time dependence of the adhesion force distribution in the parameter space of a cell population resulted in a similar spreading and widening [28]. We attribute this large variability of adhesion phenotypes to the variability of gene expression, including that of integrins and other adhesion molecules. In the future, single-cell RNA sequencing may uncover the expression profiles of cells with different levels of adhesion.

## 4. Discussion and Outlook

In this study, we demonstrate the capabilities of the computer-controlled micropipette to be used as a live cell assay determining the dissociation constant (*K_d_*) of the integrin-RGD-binding in living HeLa cells without employing any labeling. Adhesion distributions were measured on surfaces with tuned RGD densities. We demonstrated that the overall adhesion of the population could be quantified through the weighted average of the distributions. Fitting of the chemical law of mass action resulted in K_d_^2D^ = 4503 ± 1673 1/µm^2^ from which K_d_^3D^ = 74 ± 28 µM was calculated.

The acquired value was verified by applying the established waveguide-based biosensor assay to measure the equilibrium dissociation constant on the same system, which resulted in a value of K_d_^2D^ = 8433 ± 1679 1/µm^2^ thus K_d_^3D^ = 140 ± 28 µM. Binding assays using free integrin solutions reported *K_d_* = (0.089–10) µM depending on the integrin subtypes. It is expected that live cell-based methods show a different *K_d_* since these measurements take into account a wide range of integrin subtypes expressed in the cell membrane. These subtypes, in certain cases, have the ability to regulate each other (integrin crosstalk), making a direct comparison with studies using soluble integrin difficult [13,21].

Dissociation constant values acquired by the micropipette and the RWG biosensor reasonably match, corroborating the ability of CCMP to be used as a quantitative live-cell adhesion assay.

While retaining the ability to collectively characterize cell populations, the micropipette-based method is capable of additionally identifying subpopulations based on the adhesion phenotype. These subpopulations could then be examined as a function of the external condition (the RGD density in our case). It was shown that weakly adhered cells constitute a significant part of the population, even at high RGD densities. The change of the adhesion distribution as a function of RGD density was shown to be dominated by spreading and not the translation of the histogram.

The described method is specially adapted to determining the specific reactions of adhesive subpopulations to changing conditions or treatments.

## Data Availability

The data presented in this study are available on request from the corresponding author.
